# Classifying development stages of primeval European beech forests: is clustering a useful tool?

**DOI:** 10.1186/s12898-018-0203-y

**Published:** 2018-11-20

**Authors:** Jonas Glatthorn, Eike Feldmann, Vath Tabaku, Christoph Leuschner, Peter Meyer

**Affiliations:** 1 Plant Ecology and Ecosystems Research, University of Goettingen, Untere Karspüle 2, 37073 Goettingen, Germany; 2 Silviculture and Forest Ecology of the Temperate Zones, University of Goettingen, Büsgenweg 1, 37077 Goettingen, Germany; 3 Chair of Silviculture and Forest Ecology, Agricultural University of Tirana, Tirana, Albania; 4 Sachgebiet Waldnaturschutz/Naturwald, Nordwestdeutsche Forstliche Versuchsanstalt NW-FVA, Grätzelstrasse 2, 37079 Goettingen, Germany

**Keywords:** *Fagus sylvatica*, Forest dynamics, Spatial observation scale, Moving window, Primeval forests, Forest development cycle

## Abstract

**Background:**

Old-growth and primeval forests are passing through a natural development cycle with recurring stages of forest development. Several methods for assigning patches of different structure and size to forest development stages or phases do exist. All currently existing classification methods have in common that a priori assumptions about the characteristics of certain stand structural attributes such as deadwood amount are made. We tested the hypothesis that multivariate datasets of primeval beech forest stand structure possess an inherent, aggregated configuration of data points with individual clusters representing forest development stages. From two completely mapped primeval beech forests in Albania, seven ecologically important stand structural attributes characterizing stand density, regeneration, stem diameter variation and amount of deadwood are derived at 8216 and 9666 virtual sampling points (moving window, focal filtering). K-means clustering is used to detect clusters in the datasets (number of clusters (k) between 2 and 5). The quality of the single clustering solutions is analyzed with average silhouette width as a measure for clustering quality. In a sensitivity analysis, clustering is done with datasets of four different spatial scales of observation (200, 500, 1000 and 1500 m^2^, circular virtual plot area around sampling points) and with two different kernels (equal weighting of all objects within a plot vs. weighting by distance to the virtual plot center).

**Results:**

The clustering solutions succeeded in detecting and mapping areas with homogeneous stand structure. The areas had extensions of more than 200 m^2^, but differences between clusters were very small with average silhouette widths of less than 0.28. The obtained datasets had a homogeneous configuration with only very weak trends for clustering.

**Conclusions:**

Our results imply that forest development takes place on a continuous scale and that discrimination between development stages in primeval beech forests is splitting continuous datasets at selected thresholds. For the analysis of the forest development cycle, direct quantification of relevant structural features or processes might be more appropriate than classification. If, however, the study design demands classification, our results can justify the application of conventional forest development stage classification schemes rather than clustering.

**Electronic supplementary material:**

The online version of this article (10.1186/s12898-018-0203-y) contains supplementary material, which is available to authorized users.

## Background

In primeval and old-growth European beech forests (*Fagus sylvatica* L.), stand replacement is mostly not caused by large disturbances like fire, severe windthrow or insect calamities. Instead, natural regeneration often takes place on a small scale initiated by the age-related dieback of single old trees leading to the formation of small gaps of ca. 100–250 m^2^. Subsequently, groups of saplings and young trees start to develop [1]. Advanced regeneration beneath the canopy of old trees is also frequently observed [1, 2] and cover considerable portions of gap area at the time of gap formation [3]. Without human influence, it is thought that European beech forests would represent multi-cohort forests on a small scale. It is, however, a matter of debate to which extent also large infrequent disturbances are driving stand dynamics and which area typically is covered by single-cohort patches with more or less homogeneous structure.

Usually there is no information on the true age of trees in primeval beech forests. As a consequence of their multiple-cohort structure and the resulting complex individual growth patterns [2], “stand age” (i.e., the time since the last larger disturbance event) is not an appropriate attribute to characterize the development status of a certain patch of primeval beech forests, as the age of trees in primeval forests varies on small spatial scales [4]. Instead, classification into development stages and further subdivision into development phases of the forest development cycle [5] has been introduced by Leibundgut [6] and Korpeĺ [1] for European primeval forests and is widely accepted as a surrogate for stand age. Based on this categorization, different models have been developed to describe natural forest dynamics over time (e.g., [1, 7–9]). Oliver and Larson [10] distinguish four different development stages for single- or multiple cohort stands: (1) the stand initiation stage occurs when a disturbance event causes partial or complete breakdown of the overstory; (2) during the stem exclusion stage, competition is the main cause for mortality and stem number continuously decreases while living biomass is accumulating; (3) in the understory re-initiation stage, more light may reach the ground when suppressed trees die and tree saplings and small trees establish; and (4) during the old-growth stage, large and senescent trees die and small to medium sized gaps form which are rapidly filled again by lateral branch growth of neighboring trees or by understory trees. In multiple cohort stands like primeval beech forests, cohorts in all of these stages may occur simultaneously and horizontally layered.

In forest ecosystem research, the concept of forest development stages is used among others for describing habitat quality for different organism groups [11–14] or for characterizing the development of important stand properties such as leaf area index or structural diversity [15]. While it is convenient to describe and classify growth phases of single trees (e.g., through age or diameter classes) and to distinguish development stages of single-cohort stands, classification of multiple-cohort stands is much more difficult. In the past, distinction between development stages was mostly done with dichotomous keys which use thresholds of specific stand structural attributes at the plot level (SSA, for example basal area, height or amount of deadwood) [7, 8, 14, 16]. Recently, with the aid of computer algorithms, more sophisticated classification methods for development stages and phases were developed [17–19].

All these methods have in common that a priori assumptions about development stages and their characteristic compositions with respect to the used SSAs are made. For example, the occurrence of a certain amount of deadwood is usually one of the criteria for a forest patch to be assigned to the terminal development stage (senescence, breakdown stage [1, 17]). These approaches with parameter delimitation based on expert opinion neglect the possible existence of biologically-determined thresholds in the structural data of old-growth forests, which could mark the transition from one development stage to another. Such breakpoints might occur if SSAs do not change gradually over time but when the stand structure adapts more rapidly after certain SSA thresholds are reached and/or discrete disturbance events change the intrinsic development. For example, the diameter distribution of some primeval European beech forests peaks at mid-range diameters at breast height (DBH) [20]. This may indicate pulses of tree establishment caused by past disturbances. Another explanation is that trees reach the upper canopy at these DBH-classes which reduces competition with larger individuals and mortality rates drop immediately at such a site-specific diameter threshold [20]. We assume that similar effects can be observed and are more pronounced when multidimensional datasets of the stand structure (one dimension for each included attribute) of primeval forests are analyzed. Our hypothesis is that multidimensional point clouds of such data-matrices from primeval forests are not homogeneously distributed, but that spatially separable clusters do exist which are corresponding to the development stages of the natural forest development cycle.

To test this hypothesis, we use stand structural data from two completely mapped primeval beech stands in Albania: Mirdita and Rajca. A moving window (focal filter) approach is used to aggregate the SSA-data in virtual plots over the entire area of two forests. Two parameters of the moving window (virtual plot size and kernel) were varied in a sensitivity analysis to ensure that potentially existing effects are not missed because of an inappropriate study design. We then applied an unsupervised classification algorithm (k-means clustering) to detect potentially occurring clusters in the stand structural dataset without making a priori assumptions about potential thresholds. According to our hypothesis, potentially occurring clusters would correspond with the stages of the natural forest development cycle. K-means clustering requires the pre-specification of the number of clusters k. To avoid assumptions about the number of occurring stages, we used different values for k (2, 3, 4, and 5).

Our results may help to understand the fundamentals which form the basis of the classification of forest development stages and phases. We are asking the question: Does the multidimensional distribution of a primeval forest SSA-data matrix show a clustered configuration with clearly distinguishable thresholds between clusters? If so, we assume that such thresholds mark site-specific transitions from one development stage to another. Such thresholds would represent prime candidates to be used in site-adapted classification schemes. If no clustered configuration appears, this would point towards a rather homogeneous and continuous distribution of the SSA data-matrix in higher dimensional space, indicating the lack of predetermined thresholds. In that case, the selection of thresholds based on ecological theory and by expert opinion would be justified.

## Results

### Emergence of clusters at different observation scales

**Fig. 1:**
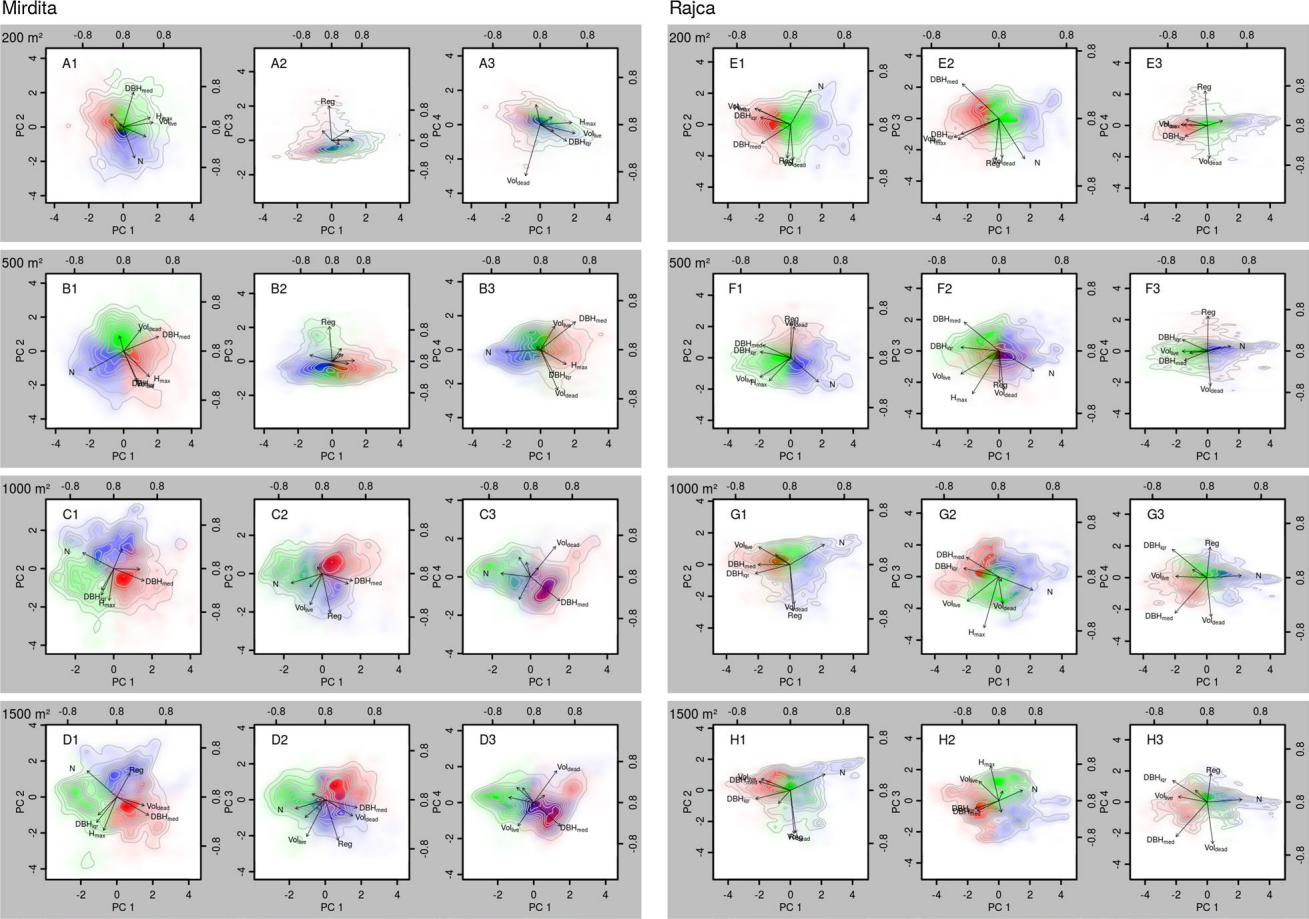
Biplots of the first principal components (PC) of inventory data of two primeval beech forests (7 attributes, see Table 2). The colored area and the contour lines represent PC-scores. Arrows depict PC-loadings. A moving window was used to aggregate the datasets from completely mapped data at four observation scales (window sizes; separated by grey shaded areas). Contour lines mark areas with equal point densities (in total 8216 points in Mirdita and 9666 points in Rajca). Coloring represents the results of k-means clustering with 3 clusters (red, green and blue; color mixing indicates overlap of two or more clusters). A uniform kernel was used for the moving window (equal weighting of all objects within the window). For results of a bivariate normal kernel (weighting of objects by their distance to the window center) see Additional file 1: Figure S1

In Fig. 1 the first principal component (PC) of the aggregated SSA data-matrices is plotted against the second to fourth principal component and results of k-means clustering (k = 3) are depicted. In the Mirdita site small observation scales (virtual plot sizes of 200 m^2^ and 500 m^2^) lead to a uniform and homogeneous point distribution of the SSAs (panels A1 to B3). No obvious groups are visible, and k-means clustering leads to an arbitrary division of the point clouds. With an observation scale of 1000 m^2^, a vague structure is emerging. At least two bigger clusters are visible when PC2 or PC3 is plotted against PC1 (panels C1 and C3). When looking at PC3 and PC1 (panel C2) several smaller subclusters are apparent as well. All clusters are not clearly distinguished from one another but blurring at their borders. At the 1500 m^2^-scale the image is similar as at the 1000 m^2^-scale with two main clusters and several smaller and blurring subclusters. Regardless of the number of clusters used for the clustering algorithms, the clustering quality did not change. No optimal number of stages to describe the forest development was detected.

In the Rajca site the trend of a somewhat better discriminability with increasing plot size is visible as well but clusters do only emerge at the largest scale of 1500 m^2^ (panels H1 to H3). At smaller scales (panels E1 to G3) some peaks are visible (e.g., panels F1 to G2), but possible clusters are not very well distinguished.

The bivariate normal kernel provides a very similar picture as the uniform kernel (Additional file 1: Figure S1). The overall appearance of the contour plots of the principal component scores generated by the normal kernel is a bit smoother with a less ragged shape of the point clouds, but clusters are not better distinguishable from each other.

### Quality of the clustering

Average silhouette widths obtained for the clustering of the SSAs by this study were mostly below 0.25 (Table 1). Silhouette widths typically lie between 0 and 1, small negative values are possible as well. Values close to one imply a strong structure in the data. Average silhouette widths smaller than 0.25 are indicative for `no substantial structure’ according to [21]. There was a slightly better separability of the datasets at the greater observation scales in Mirdita with a maximum value of 0.27 of the 5-cluster solution at the 1500 m^2^-scale. Except for that, the cluster solutions did have an equally low quality for all observation scales, both kernels and study areas.

**Table 1:** Average silhouette widths of the clustering solutions of stand structural data of two primeval beech forests

		Uniform kernel				Bivariate normal kernel			
		Number of clusters				Number of clusters			
		2	3	4	5	2	3	4	5
	Observation scale								
Mirdita	200 m^2^	0.18	0.16	0.18	0.22	0.17	0.17	0.21	0.22
	500 m^2^	0.18	0.19	0.21	0.22	0.18	0.18	0.21	0.17
	1000 m^2^	0.20	0.22	0.24	0.24	0.19	0.21	0.23	0.23
	1500 m^2^	0.20	0.26	0.26	0.27	0.20	0.24	0.23	0.25
Rajca	200 m^2^	0.22	0.19	0.22	0.23	0.21	0.21	0.23	0.22
	500 m^2^	0.21	0.23	0.20	0.20	0.21	0.23	0.18	0.20
	1000 m^2^	0.21	0.18	0.20	0.20	0.21	0.18	0.21	0.21
	1500 m^2^	0.21	0.22	0.24	0.21	0.21	0.19	0.23	0.23

A moving window approach with a uniform and a bivariate normal kernel and of several observation scales (rows) was used. K-means clustering (k = 2 − 5, columns) was applied to obtain different clustering solutions

### Between-cluster differences in stand structural attributes

**Fig. 2:**
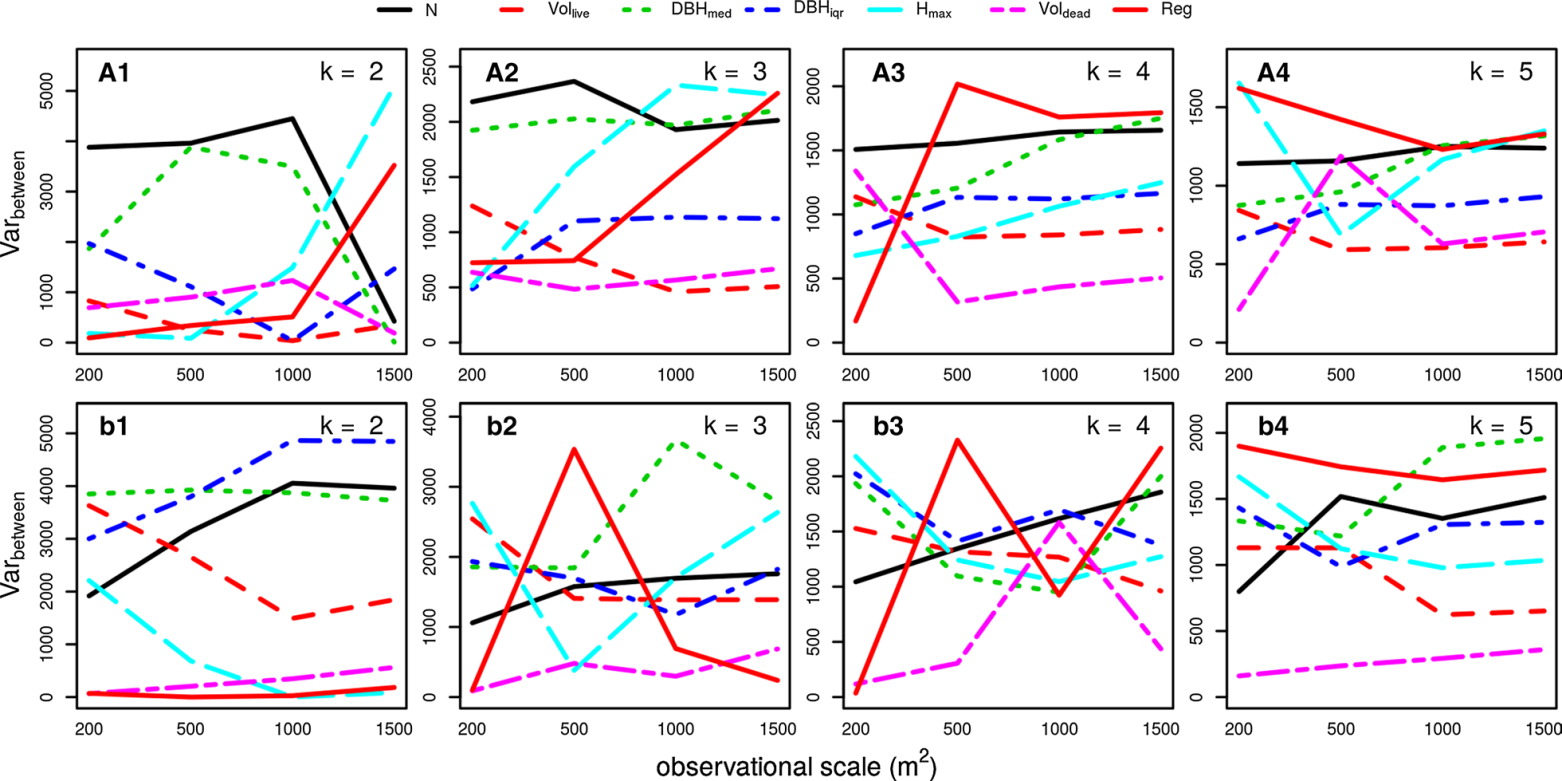
Between-clusters variance of stand structural data (7 attributes, abbreviations see Table 2) of the primeval beech forests Mirdita (**A1**–**A4**) and Rajca (**B1**–**B4**). K-means clustering was used to detect clusters (2 to 5 clusters, panels 1 to 4). A moving window approach with a uniform kernel (equal weighting of all objects within the window) of several observation scales was used to aggregate the datasets (X-axis). For the results of a bivariate normal kernel see Additional file 2: Figure S2 (weighting of objects by their distance to the window center)

The relevance of each SSA for the specific clustering solution was analyzed with the between-cluster variances of the standardized SSA (Var_between_, Fig. 2 for the data aggregated with the uniform kernel and Additional file 2: Figure S2 for the bivariate normal kernel). In Mirdita, N and DBH_med_ (negatively correlated, see loadings of the principal components in Fig. 1) were of higher relevance than the other attributes for most of the cluster solutions. Other important factors were H_max_ and V_live_ (positively related), whereas the other attributes (V_dead_, Reg) were relevant for only some spatial scales and clustering solutions. In Rajca, the most relevant attribute changed a lot between observation scales and cluster number. Even though there was no single attribute and no set of combined attributes which was most important for the determination of all clusters, differences between V_dead_ of the clusters were almost always only minor. This attribute seemed to have negligible relevance for most clustering solutions.

### Spatial representation of clusters

**Fig. 3:**
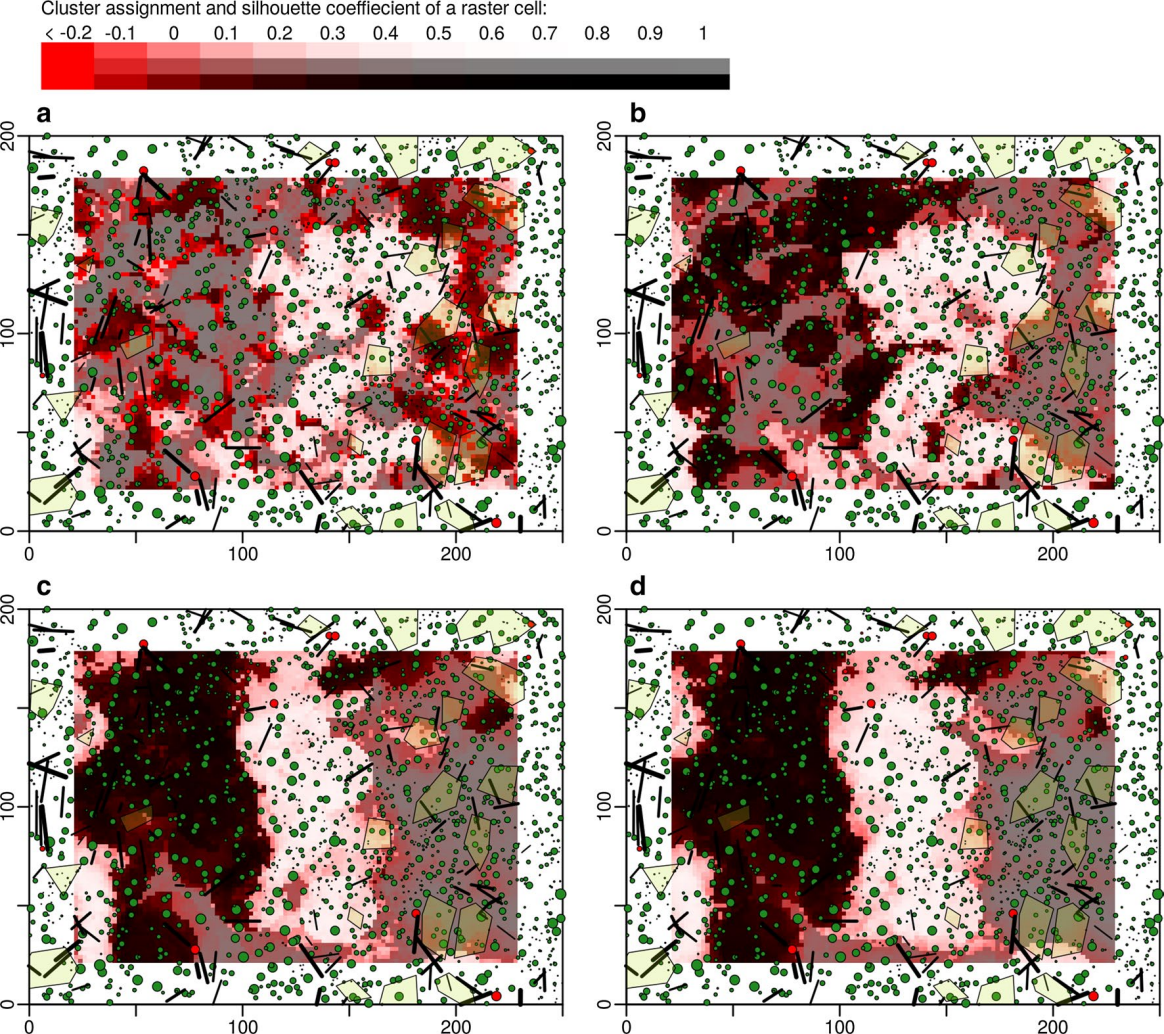
Stem position maps of the primeval forest Mirdita with k-means clustering solutions of the structural data highlighted (3 clusters). Coloring of the background images indicates areas which were assigned to the same cluster (gray tone) and how well a point is represented by its cluster (silhouette coefficient, red tone). A moving window approach of several observation scales (200 m^2^, 500 m^2^, 1000 m^2^, 1500 m^2^, panels **a** to **d**) was applied to aggregate the structural datasets (7 attributes, Table 2) which was used by the clustering algorithm A uniform kernel was used for the moving window (equal weighting of all objects within the window). For results of a bivariate normal kernel (weighting of objects by their distance to the window center) see Additional file 3: Figure S3

Maps of areas with similar stand structure differed greatly between the observation scales (Fig. 3 and Additional file 3: Figure S3). At low observation scales (200 m^2^ and 500 m^2^, panels A and B) the mosaic-like structure of patches with a homogeneous stand structure belonging to different clusters was more fine-grained with patch sizes often below 100 m^2^ (panels A and B). Single, dominant features within the borders of a virtual plot often decided about the clustering outcome. But even at such small scales, large patch sizes stretching over 1 ha and more occurred. Many of the patches touched the outer limits of the study area, so their absolute size is unknown. To accurately estimate patch-size distributions, the study area size would have to be several times larger.

At greater observation scales (1000 m^2^ and 1500 m^2^, panels C and D), the overall appearance of the patch distribution was a lot smoother. Patch sizes were bigger and often stretched over 200 m^2^ and more. Single features did not dominate the clustering process anymore.

**Fig. 4:**
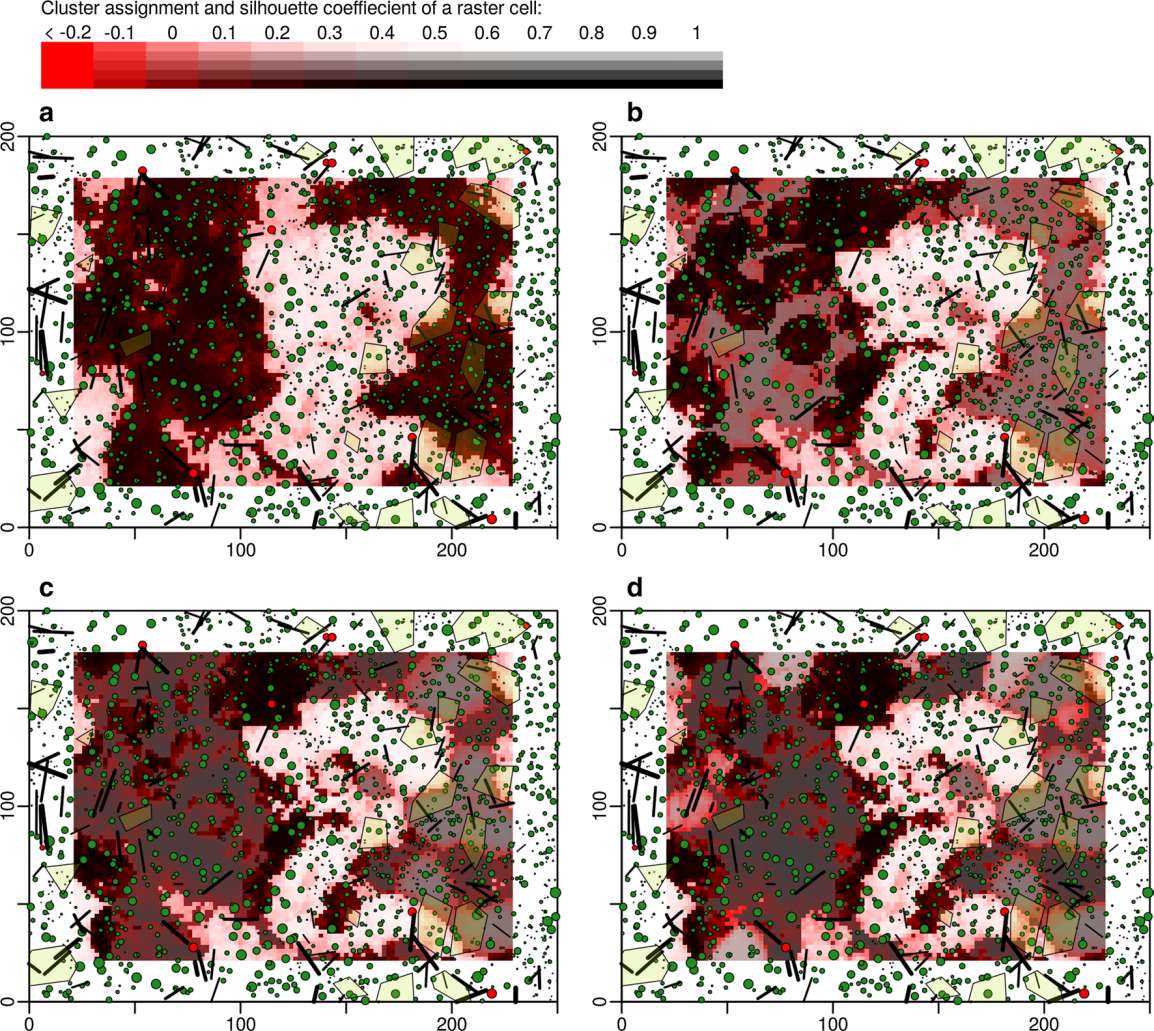
Stem position maps of the primeval forest Mirdita with k-means clustering solutions of the structural data highlighted (2 to 5 clusters, panels **a** to **d**). Coloring of the background images indicates areas which were assigned to the same cluster (gray tone) and how well a point is represented by its cluster (silhouette coefficient, red tone). A moving window of an observation scale of 500 m^2^ was used to aggregate the structural datasets (7 attributes, Table 2). A uniform kernel was used for the moving window (equal weighting of all objects within the window). For results of a bivariate normal kernel (weighting of objects by their distance to the window center) see Additional file 4: Figure S4

Maps of the same observation scale but with differing numbers of clusters produced similar results (Fig. 4 and Additional file 4: Figure S4). When the number of clusters was increased, usually one cluster was split instead of creating a completely new classification of the points. The maps in Fig. 4 depict areas with homogeneous stand structures at a specific observation scale.

At all observation scales, the silhouette coefficient (red shade of pixels) was biggest in the areas close to patch borders. These horizontal transition zones between homogeneous forest patches were particularly hard to classify for the clustering algorithm. In the center of patches, areas with low silhouette coefficients were less frequent but did occur as well.

## Discussion

The visualization of potentially existing clusters in the stand structural data with the first principal components did not reveal substantial aggregation of data points. On the contrary, only at greater observation scales of 1000 m^2^ and 1500 m^2^ there were only slight peaks visible in the contour plots (Fig. 1 and Additional file 1: Figure S1). However, the low averages of the silhouette coefficients of all cluster solutions irrespective of observation scale indicate that these peaks are no evidence of the presence of real clusters in the data. The slight peaks appearing at greater observation scales might well be just an artifact of the size of the study areas and the high similarity of points which are located close to one another. Additionally, even though both completely mapped areas were large (5 ha and 6 ha), it is likely that some common combinations of SSAs did just not occur within the boundaries of the study sites and are underrepresented in the datasets. These findings are consistent regardless of the number of distinguished clusters. No optimal number of development stages which should be used for the characterizing the natural forest development could be detected. It is unlikely that the use of a larger amount of clusters than five would have resulted in different results with improved clustering quality.

The low discriminability of the data into clusters may also be caused by a more complex stand structure of beech primeval forests than anticipated by the model of cyclic, regularly returning development stages [19, 22]. The stochastic nature of tree growth and mortality and prevalence of site specific disturbance regimes may lead to acyclic, unexpected transitions between stages [22]. Such random processes are probably of high relevance for the formation of the stand structure and may result in a high, unpredictable variation of structural attributes on small spatial and temporal scales. Even though these authors [22] do not question the overall usefulness of the concept of development stages, their results suggest that for some applications, the concept may be too simplistic. This is supported by recent analyses of gap dynamics in natural beech forests [3, 23, 24] which suggest that disturbance events of varying intensities are frequently occurring and can cause complex dynamics which are difficult to capture with the classic concept of forest development stages. In forests, where major disturbances lead to single-cohort structures, potentially existing clusters might have higher average silhouette coefficients and less blurring clusters than are observed in our forests.

The low quality of the clustering solutions suggests that, at least with respect to the studied variables, the natural forest development cycle does not lead to the emergence of clear thresholds in the SSA data-matrix between different development stages. This does not challenge the concept of classifying research plots into development stages in general, which has reliably and successfully been used to describe the forest development cycle in many previous studies (e.g., [11–13, 16, 25]). Instead, the results suggest that transitions between single stages are rather continuous. This is in agreement with other studies about the forest development cycle which show that patches of the same development stage or stand structure may take different pathways with gradually and continuously diverging composition of stand attributes [22, 26]. This supports the hypothesis of a continuous forest development life cycle as for example formulated by [7, 27]. On the one hand, this illustrates limitations of the classical approach of splitting the forest development cycle into discrete stages, as this approach may not reflect the complexity of natural forest development. This highlights the importance of analyzing single processes of forest development such as gap formation or regeneration in the course of time as done for example by [3, 28]. On the other hand, such a classification approach has proven to be a useful tool in biodiversity studies, in which it is necessary to stratify larger areas of forest according to tree age or other structural features when designing the study and analyzing the data (e.g., [12, 13]). Thus, the commonly applied practice to select thresholds based on expert opinion, which fit best to the respective ecosystems, study designs and questions, has its justification and will continually be used in future.

Even though the selected attributes to describe the forest structure in this study were selected with care, more suitable variables to reflect the natural forest development cycle may exist. In compliance with current methods to describe development stages in the field, we used state variables like stand density, maximum tree height, regeneration abundance, and others (Table 2). Ecosystem processes and functions like mortality or biomass accumulation and decay are not included neither in our selection of variables, nor in most other empirical studies addressing the classification of forest development stages. This is not because the importance of processes for forest development is neglected (in fact many authors state their relevance, e.g. [1, 10, 25]), but rather because such variables are much more difficult to monitor and suitable datasets for such analyses hardly exist. We cannot rule out the possibility that including other variables, especially variables describing ecosystem processes and functioning, might have resulted in clearer clustering solutions than observed here. However, the assessment of ecosystem processes such as productivity or decay rates typically require measurements in a high temporal resolution and are time- and labor-intensive. Until now, this prevented their consideration in classification approaches of the forest development cycle.

**Table 2:** Descriptions and abbreviations of plot-level stand structural attributes (SSA)

Abbr.	Description
N	Number of trees per hectare
DBH_med_	Median diameter at breast height
DBH_iqr_	Interquartile range of diameter at breast height
H_max_	Maximum tree height
Vol_live_	Volume of living trees per hectare
Vol_dead_	Deadwood volume per hectare
Reg	Proportion of the area covered by regeneration

**Fig. 5:**
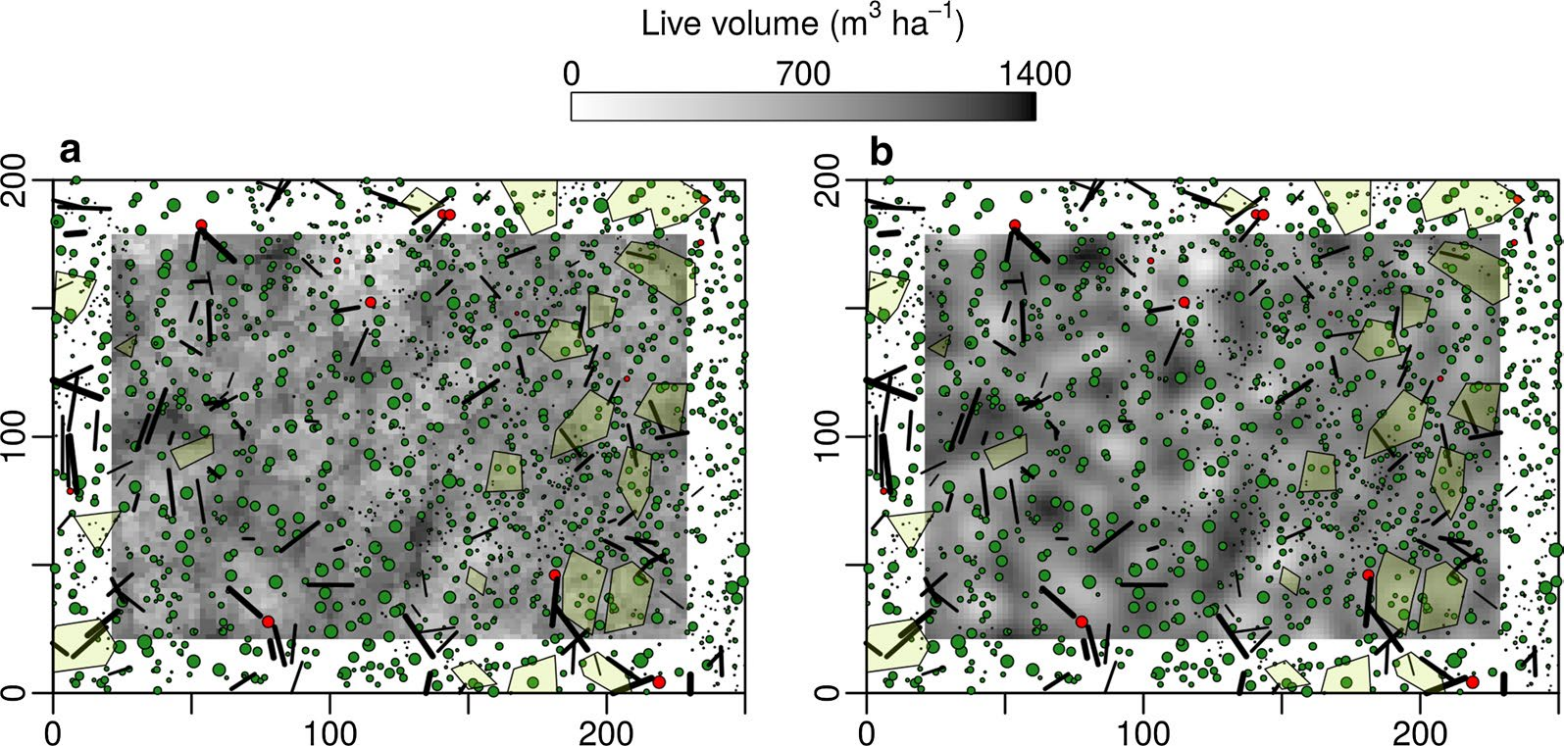
Stem position maps of the primeval forest of Mirdita. Circles mark coordinates of standing trees (green: alive; red: dead; radii proportional to diameter). Lines represent logs and green shaded areas outline regeneration patches (areas with a dense cover of trees with a diameter at breast height < 7 cm). The background raster images show results of a moving window (living tree volume) for an observation scale (window area) of 500 m^2^. **a** Results of a uniform kernel (equal weighting of all objects within the window), while for **b** a bivariate normal kernel was applied (weighting of objects by their distance to the window center)

When using the uniform kernel (i.e. equal weighting of objects within a virtual plot) for the aggregation of the structural data of the forests, the resulting maps of the distribution of the SSA over the study area displays a grainy pattern (Fig. 5a). Points lying directly next to each other (2 m distance) can be largely different depending on whether a single prominent structural feature located close to the plot border, falls within the boundaries or not. Highest aggregated values of some SSAs (e.g., live wood volume; Fig. 2) do not occur in the direct vicinity of single objects with high attribute values, but right in between two prominent objects. This is because the distribution of large trees is usually not random due to the influence of competition, but tends to be more regular at scales where competition between single trees dominates the spatial distribution of large trees (ca. 10 m and less; [29]). It is more likely to encounter multiple prominent objects within a virtual plot when its center is close to plot radius distance from such an object.

Effectively, the normal kernel aggregates attributes by calculating a weighted sum of all objects with weighting factors declining with distance from the plot center (see “Methods” section for details). This results in smoother distribution maps of the SSA when the normal kernel is applied with maximum aggregated values close to prominent objects (Fig. 5b). For the analysis of the relationship between structural features and other ecosystem attributes like regeneration or herb cover, the normal kernel could turn out to be superior. For example, when two bigger trees are located at opposite sides of a plot, half of their canopies and root systems reach beyond the plot border. Their influence in terms of light interception or water uptake at plot level is likely lower at the plot level than that of a single tree standing close to the plot center. The normal kernel accounts for this by assigning a higher weighting factor to the tree in the plot center.

In contrast to our hypothesis, the use of a bivariate normal kernel for data aggregation did not improve the performance of the clustering algorithm. Silhouette coefficients of the cluster solutions and visual appearance of the contour plots were equally poor. No otherwise hidden clusters or relationships between attributes did emerge when features more distant from a location in a primeval forest were downweighted for the assessment of its stand structure. All sort of different combinations of SSAs are equally likely and no clusters are present in the data structure. This does not discard the use of a bivariate normal kernel instead of a uniform kernel in general for the description of forest structure. For other applications, this approach still might be appropriate.

In many clustering solutions DBH_med_ and stem density N seemed to be important attributes for the separation of the clusters. As both of these variables are negatively correlated to each other (high N being associated with a low DBH_med_ and vice versa), they form one of the primary factors which were used by the clustering algorithm to differentiate between stages. The on average second most important factor used by the clustering algorithm was H_max_ together with V_live_ (which are positively related to each other). Both of these factors were arranged nearly orthogonal to each other in our study. This corresponds well with the selection of variables by [17] who used the distribution of N and basal area of live and dead trees to train a supervised classification algorithm. Other studies (e.g. [8] or [14]) only used maximum DBH, which is related to H_max_. Since the cited studies used some additional variables that have not been addressed by us (e.g. canopy cover), this may explain the missing consideration of N in these classification scheme.

The two trajectories of forest development identified in our study (N/DBH_med_ and V_live_/H_max_) are consistent with the framework developed by [10]. Low N and V_live_ values correspond to the stand initiation stage, high V_live_ and low to high N occur during the old-growth stage, depending on the progress of tree senescence and how much light reaches the forest floor. High N and intermediate V_live_ reflect the stand exclusion and understory reinitiation stage. A stage characterizing a gap immediately after a disturbance event with the simultaneous break down of several trees (i.e. low N and low V) before stand initiation stage is missing in [10].

The ecological interpretation of these partly deviating results on the occurrence of stages should not be overstressed. Clearly, much variation exists in the results with respect to changing orders of the most relevant SSAs, depending on the observation scale and the number of clusters, which is difficult to explain in ecological terms. As there is no consistent trend with increasing observational spatial scale or number of clusters investigated, variation seems to be random and mostly unpredictable.

Most SSAs varied, similar to Vol_live_, on small spatial scales. Such patterns are typical for the mosaic structure of primeval beech forests and a medium or large scale disturbance event does not seem likely in the recent past. However, even without major disturbances, there can be considerable change in the stand structure on a large spatial scale, as is documented for the gap area by two surveys within 10 years difference in a Slovakian primeval beech forest [3] or for tree species composition in a Slovakian mixed old-growth forest [30]. Differences between the two study areas may have been caused by slightly deviating site conditions and stand structures in Mirdita as compared to Rajca (according to [8], see Table 3). The first site has not as good site conditions with more shallow soils and a lower dominant height resulting in a lower live tree volume. There was a slightly higher stem number and relative gap area in Mirdita but a somewhat higher basal area in Rajca. These differences may have resulted in a different average composition development stages in the two study areas. Even without larger disturbance events, such variation in stand structure may be a characteristic element of primeval forests [19, 22] and thus may have resulted in different clustering solutions.

**Table 3:** Basic stand structural attributes of the two study sites [8]

	Stem density (N/ha)	Basal area (m^2^/ha)	Live tree volume (m^3^/ha)	Dead tree volume (m^3^/ha)	Dominant height (m)	Regeneration density (N/ha)	Canopy cover (%)	Relative gap area (%)
Mirdita	331	37.2	559.3	40.4	31.7	29,844	86.16	6.6
Rajca	391	43.4	807.4	86.0	38.5	19,259	91.36	3.3

Tabaku [8] categorized the stands in Mirdita and Rajca into 9 different developmental stages based on the same data set as used for this study applying a dichotomous key. The results of both classification schemes showed in many cases only a slightly higher agreement than would be expected from a random assignment of stages (analysis not shown). For example, the clustering solution with two clusters and a moving window size of 200 m^2^ in Mirdita distinguished one cluster which included 99% of the stand area covered by the category classified as “gap” by [8]. However, such a good agreement was an exception. On average, both classification schemes did not match very well. The thresholds used by [8], which are based on ecological theory and defined by expert knowledge, could not be reproduced by the clustering algorithms. This mismatch illustrates the difficulty of finding a classification scheme which does not underlie subjective assumptions but is still interpretable from an ecological perspective. As the differences between the clusters are rather small, random effects may influence the clustering solutions and a repeated run with the same approach could potentially result in clusters with different characteristics. This hampers comparability between studies and to studies which use a classical classification scheme.

The low relevance of Vol_dead_ for most of the clustering solutions is most likely because its spatial distribution across the study area is rather homogeneous and does not correspond well with the distribution pattern of other attributes. In contrast to earlier studies (e.g., [8, 17]), and in agreement with our results, [31] and [12] found that the amount of deadwood within a plot is not necessarily a good indicator for its development stage. The idea of high amounts of deadwood in the terminal stage and carry-over effects to the growth stage bases on the assumption of a strictly cyclic succession of development stages (i.e., → growth → optimal → terminal → growth → …, [1]). When tree cohorts of different ages are present at a forest patch and disturbance only causes a partial breakdown of the tree cover, transition from one development stage to any other may occur as described in depth by [22]. In conjunction with a high residual time of deadwood logs and snags up to 50 years [32], this may cause high deadwood amounts in any part of the development cycle. Deviating conclusions on deadwood persistence in the forest cycle by other studies may be caused by diverging classification methods. When the amount of deadwood is a key variable in a dichotomous key to assign development stages, conclusions about varying amounts of deadwood are circular reasoning.

At first glance, only moderate or missing relationships between the deadwood amount and forest development stage seem to be surprising because processes like mortality and decay doubtlessly play central roles in natural forest dynamics. However, the total amount of deadwood may not be a good proxy for such processes, as it just describes the status quo and not the underlying dynamics. A classification of deadwood objects into decay classes, which account for elapsed time since tree death, or direct measurement of deadwood dynamics through repeated measurements or recording of respiration rates may lead to results which are more closely linked to forest development stages.

K-means clustering does not result in clearly separated clusters with distinct thresholds. However, the algorithms still lead to the objective splitting of the study sites into zones with maximum similarity within the same zone and maximum difference to areas of other zones (Figs. 3 and 4; Additional file 3: Figure S3, and Additional file 4: Figure S4). Areas of the same cluster in the maps were more likely subject to a similar development history. Large connected areas with a homogeneous stand structure (100 m to 200 m in length) give an impression, at which scale stand replacement takes place in primeval forests.

Patch sizes obtained by this method are, irrespective of the observation scale, all larger than patches identified for example by the classification into development stages with supervised algorithms as done by [17]. Besides the effect of different computational methods, different patch sizes may also be identified because of differing stand dynamics in stands with functionally diverse tree species compositions (e.g., mixtures of broadleaved and coniferous species in spruce-silver fir-beech stands as in [17] compared to almost pure beech stands investigated here) or a differing set and weighting of specific SSAs used in the different studies. Patch size may also depend on the number of development phases distinguished. For example, [8] identified eight development phases with the consequence that observed patch size was smaller than in our study with separation of two to five stages.

## Conclusions

The evaluation of the clustering process revealed that the point clouds of the structural data are rather homogeneous without clearly separated clusters in the data of the two investigated primeval forests. The clusters do not correspond well with development stages of the forest development cycle. This implies that the maps of the clusters are as well not representing development stages. However, they successfully and precisely outline areas with highest possible similarity within and highest possible distinctness between patches of the same category. Patch size and distribution of such clustering solutions may help to assess at which spatial scales primeval forests are structured.

The low clustering quality shows that the forest development cycle is continuous and that any separation of development stages relying on stand structural data means to split a continuous point cloud. This is valid at least for primeval beech forests in Albania. These results help to better understand the procedure of forest development classification. Just as the classification of tree DBH into diameter-classes, which is a long-standing practice in forestry and forest ecology, the classification of development stages does separate a continuous multivariate point cloud of a set of SSAs of a natural forest into ecologically meaningful categories. We acknowledge that all classification schemes are a simplification of the complex processes which occur during natural forest development. Much insight could already be gained by looking separately at processes of natural forest development like gap formation, regeneration or deadwood decay in the course of time instead of lumping all attributes together in a single classification scheme. However, disciplines like biodiversity research can benefit from such a simplification as it can be used, for example, to implement more efficient sampling designs through stratification of the study area. As we could not detect clusters in the point clouds, which would have suggested the existence of naturally superimposed thresholds, the current practice of selecting such thresholds with expert knowledge or with algorithms is justified for these approaches. We further suggest to apply our approach to other well-studied primeval forests of the temperate zone to reach more general conclusions on the validity of clustering in the analysis of forest development cycles.

## Methods

### Study areas

Both study sites were located in mountainous terrain and surrounded by extensive primeval beech forests without management impact. They are composed of a patchy mosaic of gaps and areas in different stages of the forest development cycle (documented by [8, 33]).

Mirdita (5 ha, 250 × 200 m) lies in the Munella mountain range in northern Albania (41°55′ N–42°7′ N; 20°3′ E–20°15′ E). The terrain is sloping (25°–30°) and has a southeastern exposition. The soils are Cambisols with relatively high nutrient supply. There is a Mediterranean mountain climate with an annual mean temperature of ca. 6 °C, annual precipitation of ca. 2600 mm and high winter precipitation (values extrapolated from the closest weather station Domgjon at 5 km distance). *F. sylvatica* is the dominating tree species; there are minor shares of *Abies alba* Mill. and *Acer pseudoplatanus* L. The forest community can be assigned to the *Fagetum asperuletosum* association.

The study site Rajca (6 ha, 400 m × 150 m) is located in the Shebenik-Jabllanica mountain ranges in the east of central Albania (41°14′ N, 21°07′ E, 1400 m–1450 m a.s.l.). The topography is as well sloping (20°–30°) with a southwestern exposition. There is no climate station close by to extrapolate annual temperature and precipitation, but climatic conditions should be similar to Mirdita. The soil type is similar to Mirdita and the forest association is also the *Fagetum asperuletosum* with minor shares of *A. alba* and *A. pseudoplatanus.*

### Forest inventory

The forest inventory was carried out in September 1998. In both study sites, standing live and dead trees with a DBH ≥ 7 cm were inventoried. DBH, decay class of the dead trees [34], and the coordinates of each tree were recorded. The tree height of a subset (100 to 150 trees per study site) of all inventoried trees was measured; the height of the remaining trees was estimated from empirically derived relationships between DBH and stand height (stand height curves). Species identity, the coordinates of the log’s end points and the decay class of lying deadwood pieces were recorded and the log diameters measured at the middle of the log. The extension of regeneration patches (areas covered by trees with a DBH < 7 cm) was approximated by polygons and the coordinates of all corner points within the study sites were recorded. For a detailed description of the inventory’s general results see [8].

Tabaku [8] used the same dataset and a dichotomous key to classify the study area into 9 developmental phases (gap, regeneration, initial, early optimal, mid optimal, late optimal, plenter, terminal and decay). Frequency and spatial distribution of the phases showed a pattern typical for primeval beech forests with high proportions of the terminal (ca. 50%) and plenter phase (ca. 20%) (see as well [16] for a similar analysis in primeval beech forests in Slovakia).

### Calculation of stand structural attributes

We used a moving window (focal filter) approach which resulted in detailed maps of the distribution of the SSAs across the study areas (Fig. 5a). A geographic information system was used to place a regular grid of 2 m spacing over each of the study sites and virtual sampling points were established at each of the grid nodes. Seven SSAs were calculated for circular virtual plots centered at each of the sampling points (Table 2). To ensure that the boundaries of all virtual plots were located within the study areas, only sample points outside a 22 m (radius of the largest virtual plot size) wide buffer zone were used. In this way, matrices of structural data with the dimension 8216 × 7 (Mirdita) and 9666 × 7 (Rajca) were generated. We chose the attributes in Table 2 because they correspond well to variables used by previous approaches to classify forest development stages [8, 9, 18, 19, 30]. However, we did not assess the attributes separately for different diameter classes as suggested by [19] or [9]. The used SSA reflect some of the main state variables of forest development as for example described by Korpeĺ [1] or Oliver and Larson [10]. Variables describing matter fluxes or processes like productivity, decay rate or gap formation are perhaps even more important for the characterization of forest development [24, 27] than state variables and should optimally be included in classification schemes as well. However, as these variables are typically very time-consuming to measure, they are hardly ever available in datasets used for the classification of forest development stages.

Lying trees often crossed the borders of the virtual plots. To account for only partial coverage of lying trees by a virtual plot, logs were segmented into 50 cm long pieces and each segment was referred to by its center coordinates. The volume of each deadwood segment was approximated by a frustum of a cone and a correction factor depending on its decay stage was applied (1, 0.95, 0.8 and 0.5). Decay classes according to [34] were used and ranged between 1 (fresh dead) to 4 (heavily decayed). The diameters at the segment’s beginning and end were estimated from the middle diameter of the respective log and an assumed tapering of 10 mm m^−1^.

Likewise, regeneration patches were rasterized into 1 m^2^ elements to calculate the proportion of the virtual plot area covered by regeneration.

The spatial variability of SSAs changes depending on the spatial observational scale (virtual plot area around sampling points; [35]). Thus, to account for the effects of variable observation scales, the analysis was conducted at four different scales (200, 500, 1000 and 1500 m^2^.

The usual procedure of assessing the stand structure of forests is via research plots of different sizes and equal weighting of all objects within the boundaries of the plots (e.g., [36]). We hypothesized that equal weighting of all objects (uniform kernel) is not optimal because objects close to the plot border are influencing the stand structure at a specific point less than objects close to the plot center (for example because a considerable part of the canopy or root system of the trees at the plot border reaches beyond the plot borders). To test this hypothesis, we additionally used a bivariate normal (Gaussian) kernel. With the normal kernel, the attributes of objects within a plot are not merely summed up, but a weighted sum is calculated with weighting factors decreasing with distance from the plot center. This resulted in smoother maps of the spatial distributions of the SSA (Fig. 5b). The bandwidths of the normal kernels were chosen to correspond best to the dimensions of the uniform kernels: the integrated kernel density of a normal kernel equaled 0.95 within the boundaries of the respective uniform kernel.

### Graphical display and clustering of the structural data

For the graphical display of the structural data, principal component analysis (PCA) was used and the first four principal components were plotted against each other (Fig. 1). Prior to analysis, all SSAs were standardized to have zero mean and unit variance. To find potentially existing clusters in the data structure, k-means clustering with two to five clusters was applied [37]. The number of clusters (k) used by the algorithm corresponds to the number of development stages which might exist in the natural forest development cycle. A considerably higher clustering quality of one of the solutions would point towards the presence of a specific number of development stages inherent to the natural development of the studied forests.

The quality of the clustering solutions was assessed with the average silhouette width [21]. This index ranges between one (indicating that a strong structure was detected) and small negative values (indicating that no structure was detected). The index takes the means of all silhouette coefficients of all individual data points. The silhouette coefficient of a single point assesses how well it is represented by its own cluster compared to the closest neighboring cluster. This is done by relating the average dissimilarity (e.g., Euclidean distance) of a point to all objects of its own cluster to the average dissimilarity of all objects of the neighboring cluster. See [38] for details. All calculations of clusters and silhouette coefficients were done in R [39] using the package “flexclust” [40]. To analyze which SSAs were most relevant for the separation of the clusters of a specific cluster solution, the between-groups (clusters) variance of the standardized SSAs known from classical discriminant analysis was used (Var_between_; [38]). For each data point the squared distance of its group mean to the overall mean of the respective attribute is determined. Var_between_ is calculated by adding up the squared distances between the respective cluster mean and the overall mean for all data points and dividing this sum by the number of clusters minus one. A high Var_between_ of an SSA indicates that the cluster means were very different from the overall mean and therefore relevant for the discrimination between clusters.

## Additional files

**Additional file 1: Figure S1.** Biplots of principal component analyses of stand structural data aggregated with a bivariate normal kernel.

**Additional file 2: Figure S2.** Between-clusters variance of stand structural data aggregated with a bivariate normal kernel.

**Additional file 3: Figure S3.** Mapping of clustering solutions of stand structural data aggregated with a bivariate normal kernel at several observational scales.

**Additional file 4: Figure S4.** Mapping of clustering solutions with 2 to 5 clusters of stand structural data aggregated with a bivariate normal kernel.

## Abbreviations

DBH: diameter at breast height
DBH_iqr_: interquartile range of diameter at breast height
DBH_med_: median diameter at breast height
H_max_: maximum tree height
N: number of trees per hectare
PC: principal component of a PCA
PCA: principal component analysis
Reg: proportion of an area covered by regeneration
SSA: stand structural attribute
Vol_dead_: deadwood volume per hectare
Vol_live_: volume of living trees per hectare
