# Supplementary material for: Classifying development stages of primeval European beech forests: is clustering a useful tool?
Source: BMC Ecol. 2018 Nov 20;18:47. doi: 10.1186/s12898-018-0203-y (PMC6247681; doi:10.1186/s12898-018-0203-y)
Supplement: Supplementary file 1 — Additional file 1: Figure S1. Biplots of principal component analyses of stand structural data aggregated with a bivariate normal kernel. [file 12898_2018_203_MOESM1_ESM.pdf]

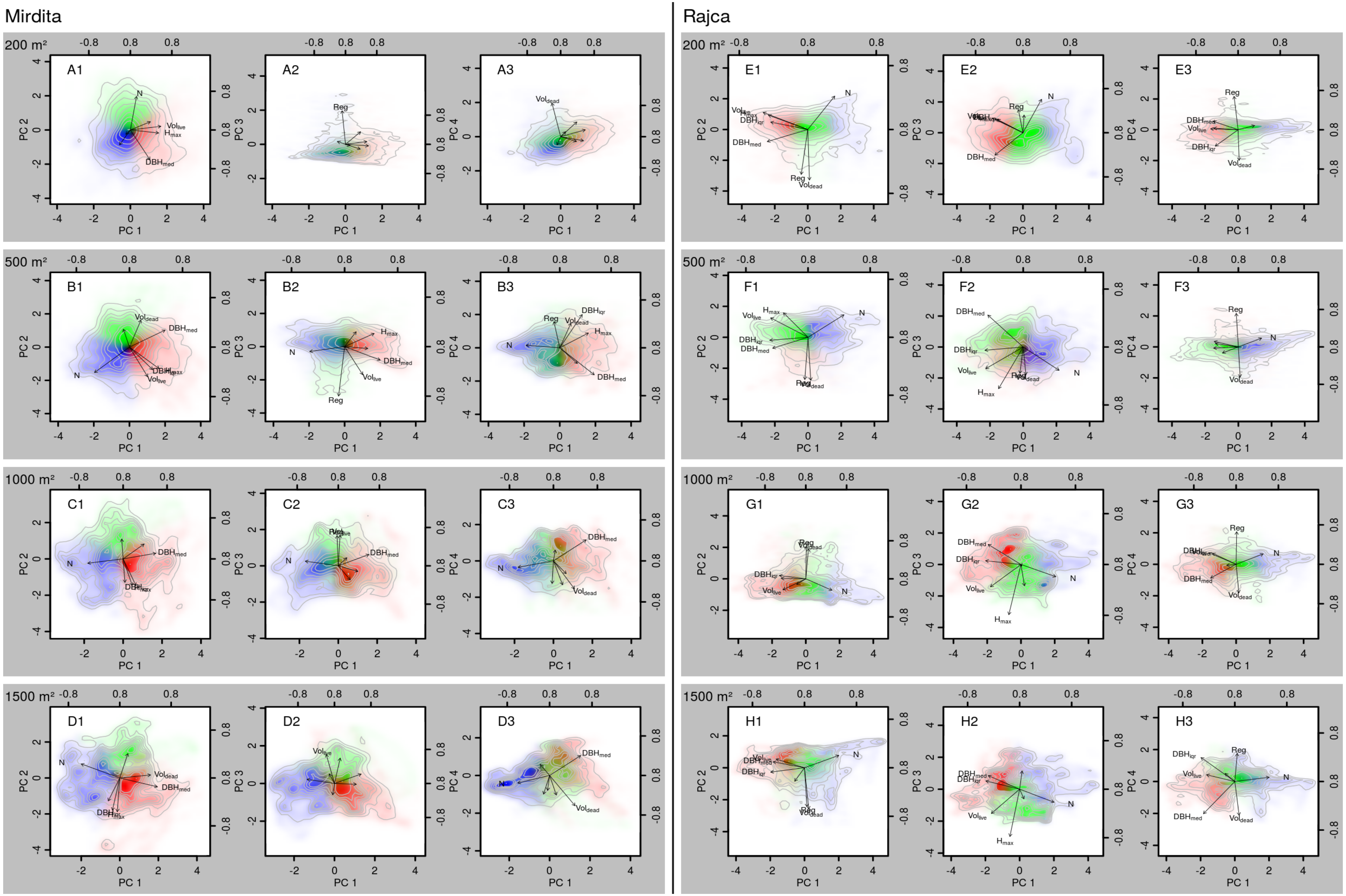

Figure S1*: Biplots of the first principal components (PC) of inventory data of two primeval beech forests (7 attributes, see Table 2). The colored area and the contour lines represent PC-scores. Arrows depict PC-loadings. A moving window was used to aggregate the datasets from completely mapped data at four observation scales (window sizes; separated by grey shaded areas). Contour lines mark areas with equal point densities (in total 8216 points in Mirdita and 9666 points in Rajca). Coloring represents the results of k-means clustering with 3 clusters (red, green and blue; color mixing indicates overlap of two or more clusters). A bivariate normal kernel was used to aggregate the data (weighting of objects by their distance to the window center).

*Additional file to: Glatthorn J, Feldmann E, Tabaku V, Leuschner C, Meyer P. Classifying development stages of primeval European beech forests: is clustering a useful tool? BMC ecology. 2018.
