# Supplementary material for: Classifying development stages of primeval European beech forests: is clustering a useful tool?
Source: BMC Ecol. 2018 Nov 20;18:47. doi: 10.1186/s12898-018-0203-y (PMC6247681; doi:10.1186/s12898-018-0203-y)
Supplement: Supplementary file 2 — Additional file 2: Figure S2. Between-clusters variance of stand structural data aggregated with a bivariate normal kernel. [file 12898_2018_203_MOESM2_ESM.pdf]

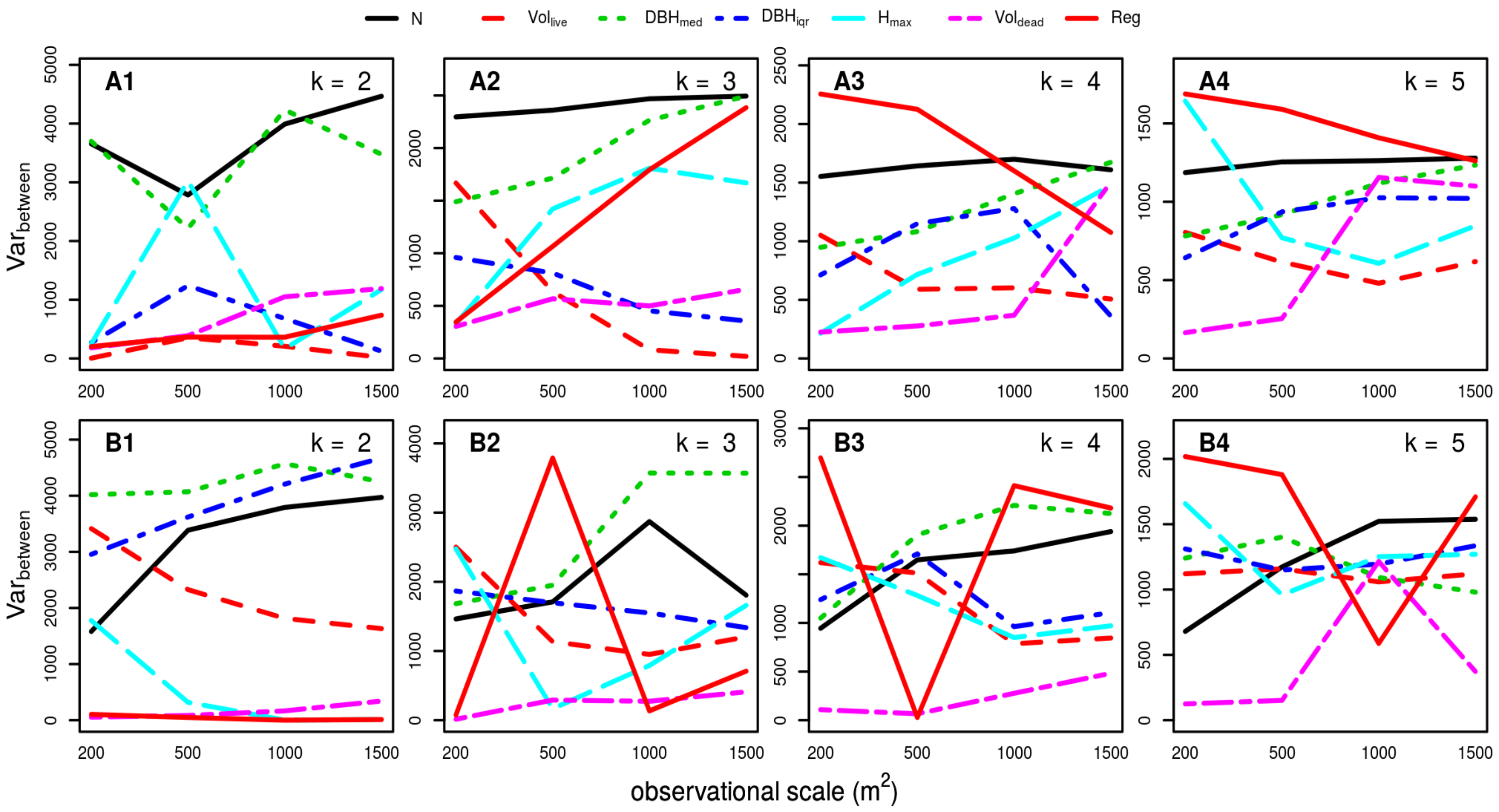

Figure S2*: Between-clusters variance of stand structural data (7 attributes, abbreviations see Table 2) of the primeval beech forests Mirdita (A1 – A4) and Rajca (B1 – B4). K-means clustering was used to detect clusters (2 to 5 clusters, panels 1 to 4). A moving window approach with a bivariate normal kernel was applied (weighting of objects by their distance to the window center).

*Additional file to: Glatthorn J, Feldmann E, Tabaku V, Leuschner C, Meyer P. Classifying development stages of primeval European beech forests: is clustering a useful tool? BMC ecology. 2018.
