# Supplementary material for: Classifying development stages of primeval European beech forests: is clustering a useful tool?
Source: BMC Ecol. 2018 Nov 20;18:47. doi: 10.1186/s12898-018-0203-y (PMC6247681; doi:10.1186/s12898-018-0203-y)
Supplement: Supplementary file 3 — Additional file 3: Figure S3. Mapping of clustering solutions of stand structural data aggregated with a bivariate normal kernel at several observational scales. [file 12898_2018_203_MOESM3_ESM.pdf]

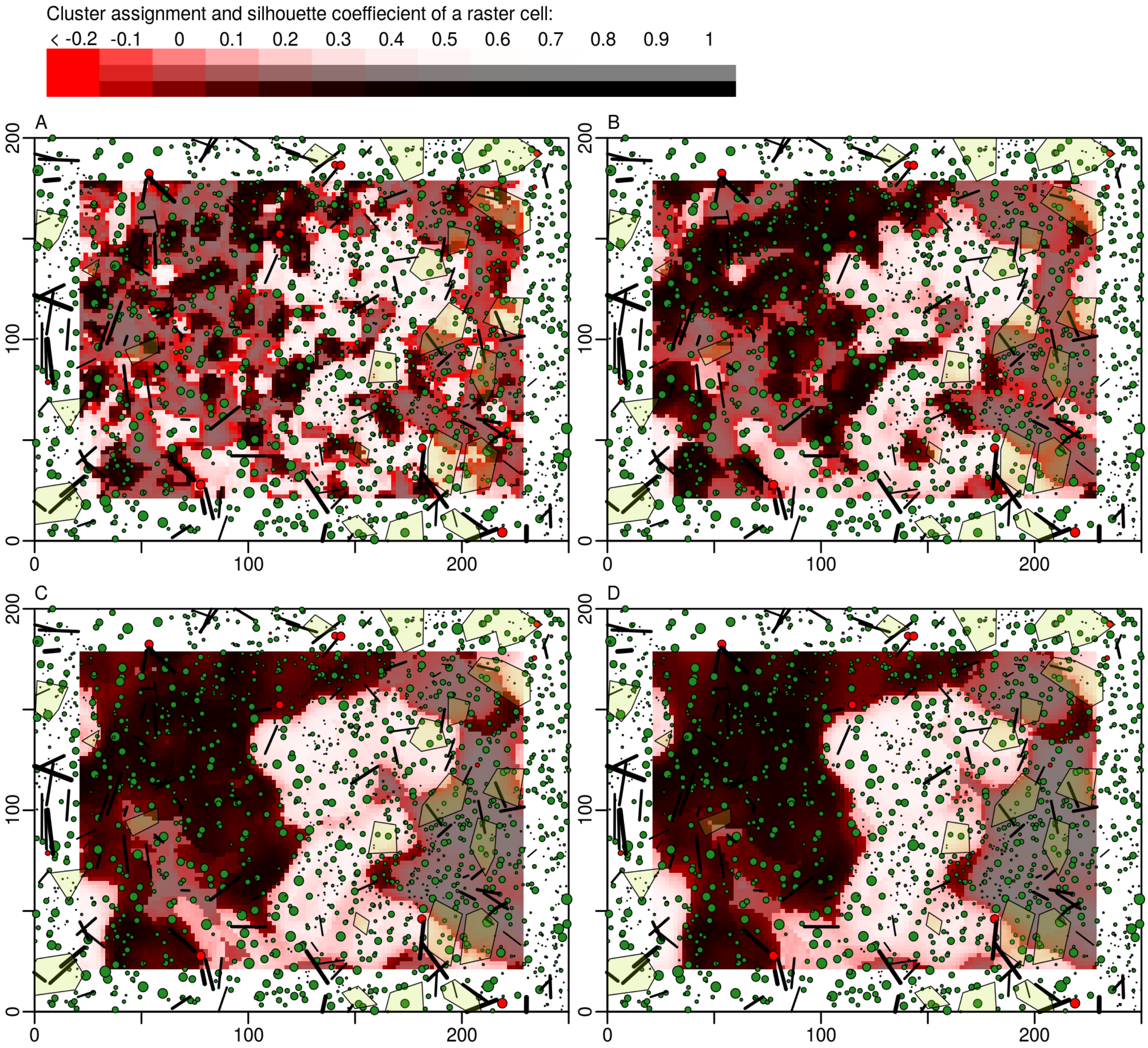

Figure S3*: Stem position maps of the primeval forest Mirdita with k-means clustering solutions of the structural data highlighted (3 clusters). Coloring of the background images indicates areas which were assigned to the same cluster (gray tone) and how well a point is represented by its cluster (silhouette coefficient, red tone). A moving window approach of several observation scales (200 m^2, 500 m^2, 1000 m^2, 1500 m^2, panels A to D) was applied to aggregate the structural datasets (7 attributes, Table 2).

*Additional file to: Glatthorn J, Feldmann E, Tabaku V, Leuschner C, Meyer P. Classifying development stages of primeval European beech forests: is clustering a useful tool? BMC ecology. 2018.
