# Supplementary material for: Classifying development stages of primeval European beech forests: is clustering a useful tool?
Source: BMC Ecol. 2018 Nov 20;18:47. doi: 10.1186/s12898-018-0203-y (PMC6247681; doi:10.1186/s12898-018-0203-y)
Supplement: Supplementary file 4 — Additional file 4: Figure S4. Mapping of clustering solutions with 2 to 5 clusters of stand structural data aggregated with a bivariate normal kernel. [file 12898_2018_203_MOESM4_ESM.pdf]

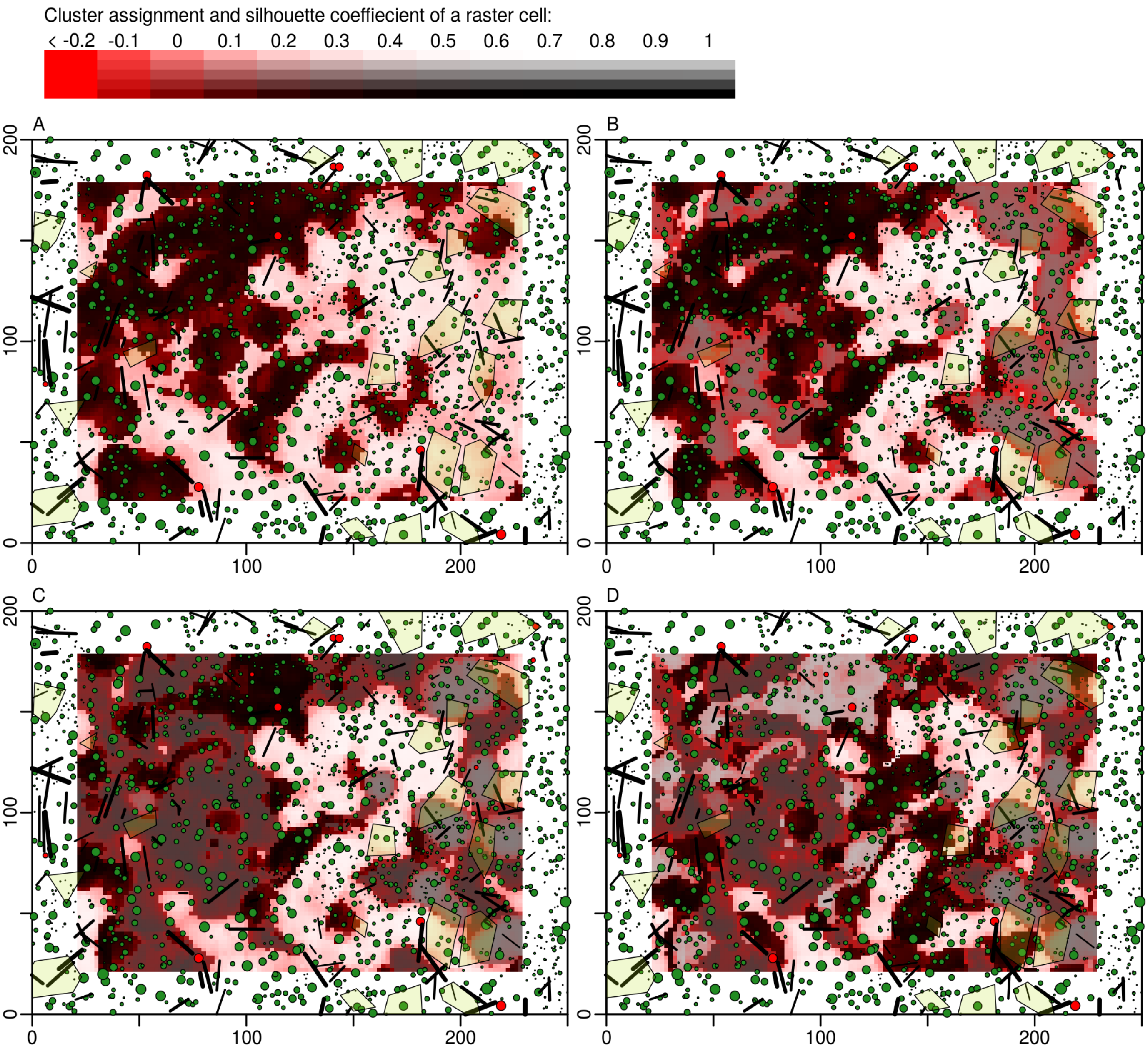

Figure S4*: Stem position maps with k-means clustering solutions of the structural data highlighted (2 to 5 clusters, panels A to D). Coloring of the background images indicates areas which were assigned to the same cluster (gray tone) and how well a point is represented by its cluster (silhouette coefficient, red tone). A moving window of an observation scale of 500 m^2 was used to aggregate the structural datasets (7 attributes, Table 2). A bivariate normal kernel was applied (weighting of objects by their distance to the window center).

*Additional file to: Glatthorn J, Feldmann E, Tabaku V, Leuschner C, Meyer P. Classifying development stages of primeval European beech forests: is clustering a useful tool? BMC ecology. 2018.
